# ‘Good’ and ‘Bad’ deaths during the COVID-19 pandemic: insights from a rapid qualitative study

**DOI:** 10.1136/bmjgh-2021-005509

**Published:** 2021-06-01

**Authors:** Nikita Simpson, Michael Angland, Jaskiran K Bhogal, Rebecca E Bowers, Fenella Cannell, Katy Gardner, Anishka Gheewala Lohiya, Deborah James, Naseem Jivraj, Insa Koch, Megan Laws, Jonah Lipton, Nicholas J Long, Jordan Vieira, Connor Watt, Catherine Whittle, Teodor Zidaru-Bărbulescu, Laura Bear

**Affiliations:** 1 Anthropology, London School of Economics and Political Science, London, UK; 2 Anthropology, Northwestern University, Evanston, Illinois, USA

**Keywords:** COVID-19, public health, qualitative study

## Abstract

Dealing with excess death in the context of the COVID-19 pandemic has thrown the question of a ‘good or bad death’ into sharp relief as countries across the globe have grappled with multiple peaks of cases and mortality; and communities mourn those lost. In the UK, these challenges have included the fact that mortality has adversely affected minority communities. Corpse disposal and social distancing guidelines do not allow a process of mourning in which families and communities can be involved in the dying process. This study aimed to examine the main concerns of faith and non-faith communities across the UK in relation to death in the context of the COVID-19 pandemic. The research team used rapid ethnographic methods to examine the adaptations to the dying process prior to hospital admission, during admission, during the disposal and release of the body, during funerals and mourning. The study revealed that communities were experiencing collective loss, were making necessary adaptations to rituals that surrounded death, dying and mourning and would benefit from clear and compassionate communication and consultation with authorities.

**Summary box**

- What counts as a ‘good death’ or a ‘bad death’ for any given community has been explored extensively in anthropological research on death, funerary rites and mourning.
- Across communities, a ‘good death’ in normal circumstances means allowing people to die in with dignity, in the company of loved ones or of others who can provide spiritual support, ensuring that their bodies undergo appropriate ritual procedures (even if the ritual has been modified for the pandemic) and respecting their wishes regarding burial or cremation.
- The challenge of how to build an infrastructure through which the bereaved were able mourn while limiting the spread of COVID-19 posed itself as a social problem for multiple stakeholders in the UK.
- The disproportionate impact of COVID-19 mortality on minority groups necessitates particular acknowledgement and memorialisation in order to prevent collective trauma and feelings of cultural exclusion.
- The use of rapid ethnographic methods to make direct recommendations for policymaking proves an effective methodology and might be pursued with respect to other issues that arise in the pandemic and its aftermath to better meet the needs of communities.

## Introduction

Dealing with excess and untimely death in the context of the COVID-19 pandemic^1^ has thrown the question of good and bad deaths into sharp relief as countries have grappled with multiple peaks of cases and mortality; and communities mourn those lost. COVID-19 fatalities exemplify ‘bad deaths’, marked by physical discomfort, difficulty breathing, social isolation, psychological distress, lack of preparation, being treated without respect or dignity, the receipt of unwanted medical interventions or being deprived of treatments desired.^2^ Pandemic control measures restricting access to hospitals and funerals have prevented patients with conditions other than COVID-19 from undergoing a normal dying process. Yet the question of how communities have responded to bad deaths with adapted processes of mourning remains under-researched, bar a number of exceptions.^3^

What counts as a ‘good death’ or a ‘bad death’ for any given community has been explored extensively in anthropological research. Such research works from the premise that death is both an instantaneous, biological end of life in the individual body and a social process of emotions and activities that severs the ties linking the deceased to the living.^4 5^ Across cultures, a ‘good death’ might involve a dignified and pain-free death, a moral obligation from the living to care for the body, a period marked by rites enabling the deceased to transition away from the world or the placing of remains in an appropriate way. As such, death is a critical event within kinship networks, where the living are unable to return to their lives, and social order cannot be restored, until an obligation is observed to death through the proper ritual acts.^6^

The handling and memorialisation of dead bodies are often most distressing in those cases where they are considered not to happen properly.^7–10^ If the proper rites are not performed, the dead may remain both vulnerable themselves and dangerous to the living. A ‘bad death’, hence, is one where the appropriate process is not followed, and where the deceased is not given the dignity she is due by the bereaved. Many examples of this have been documented including in contexts of untimely death,^11–13^ where bodies are absent,^14^ deprived of proper rites^15^ or have to be disinterred.^16 17^ Situations of excess death caused by temporary or chronic disease also produce the conditions for ‘bad death’. For instance, the Ebola epidemic has highlighted the clash between traditional/cultural and biomedical/bureaucratic ideas of a ‘good death’.^18^ The HIV pandemic has shown how excess mortality shifts the rituals and obligations that surround death.^19–21^ Such insights are useful for understanding how we might approach death in times of distress, where normal practices are not possible.^22^

In the UK, as of 25 May 2021, a total of 127 739 deaths have occurred within 28 days of receiving a positive COVID-19 test. Recent research in the USA suggests that every COVID-19 death leaves nine bereaved kin,^23^ translating in the UK to over 1 million bereaved.^24^ Yet, over the course of the pandemic, the public conversation surrounding mortality from COVID-19, as propelled by both the UK government and media coverage, has diminished. Despite data that suggest that excess death has occurred since 18 March 2020,^25^ it has been normalised in public discourse as the crisis has prolonged. There is a lack of nuance in the presentation of death and grief in the media, and a tension between sensationalist accounts and discourse which attempts to mitigate the threat of disease.^26^ In the UK, mortality has adversely affected^27^ and has been reported differently^28^ for minority groups such as Bangladeshi and Pakistani groups, and deprived communities.^29^ The fact that such excess death has occurred disproportionately exacerbates the feeling that some lives are worth more than others—producing new social divides and forms of distrust in government.^30 31^

The challenge of how to build an infrastructure wherein communities are able to mourn those who have died a ‘bad death’ while limiting the spread of COVID-19 posed itself as a social problem for multiple stakeholders. In the first weeks of the pandemic, the families of the dying were prevented from entering intensive care units, accessing bodies immediately after death and restrictions were placed on funerals.^32^ Such stipulations prevented the customary practices prior to death, in saying goodbye, funerary rituals or mourning, causing significant distress.^33^ Restricted grieving processes have been compounded by an erosion social support, including social isolation, financial precarity, uncertainty and lack of routine.^34 35^ Mourners and community leaders have, however, expressed creativity and flexibility in adapting ceremonies and mitigating distress.^36^

This study presents a reflective analysis of how death was managed in the early stages of the UK’s pandemic by drawing on ethnographic research conducted with leaders of faith and non-faith communities in April 2020. Research began by exploring how people across various faith and non-faith groups were already adapting processes of dying and responding to government regulations. It focused on five moments in the dying process—preadmission, hospital admission, body release, funerals and mourning. It suggests policies that might be proactively institutionalised in order to avoid collective trauma in future crises.

## Rapid ethnographic research

The study design was informed by precedents in policymaking that mobilise ethnographic and participatory methods to understand and meet the needs of certain communities,^37^ even in a rapidly evolving situation such as a pandemic.^38^ The strengths of ethnographic methods, even when deployed rapidly, include careful attention to the effects of the specific contexts within which policies might be embedded; connections to economic, political and historical forces; and focus on encounters with the government or health system.^39^

The study involved a rapid ethnographic exercise in which a team of 17 anthropological researchers conducted 58 interviews of 30–60 min in 1 week (3–9 April 2020) via WhatsApp, Zoom or telephone. Rather than trying to find a representative cross section of respondents as in Office for National Statistics survey data collection or citizen juries, our sampling strategy involved identifying ‘local experts’ who were at the centre of dense networks of social interaction and had access to a large amount of information on experiences changing in real time. They were recruited through the research team’s existing personal or professional networks, concentrated in London and the South East, but also spanning the East Midlands, East Anglia, Northern Ireland, Scotland and Wales. Participants were faith and community leaders, those involved in the funeral industry and in palliative care. They included representatives from various denominations of the Sikh, Hindu, Jewish, Muslim and Christian communities; and representatives of various minority communities including those from African, Afro-Caribbean, South Asian and Middle Eastern backgrounds. Participants were asked about what their community members were most concerned about in relation to death, hospitalisation and mourning, their willingness to adapt existing body disposal and congregation processes; and historical precedents where an immediate response to excess death and long-term management of trauma were necessary. Results hence focused on different community responses to situations of death in normal circumstances, and how death and mourning were managed by communities during the early stages of the pandemic. Interviewers transcribed and summarised their interviews, which were then read by the group. The research team met in a workshop where key themes were identified. One researcher (NS) coded the transcripts according to agreed themes and analysed the findings to produce a rapid report that the research team provided comments on.

## The dying process

### Anticipating death

Community leaders were encouraging their communities to show love and care for those close to them in preparation for bereavements. In some, particularly minority communities, there was some stigma surrounding contracting COVID-19, causing individuals and families to avoid revealing their symptoms or diagnosis. Many people did not have their affairs in order before they were admitted to hospital, causing distress for their family members. The period leading up to hospital admission was perceived as more distressing if patients face additional stressors such as accessing welfare support, unemployment and caring responsibilities.

### Hospitalisation

Community leaders perceived that, for the National Health Service (NHS), safe death was understood as one where those in overburdened intensive care units were not visited, and life support was ceased at an appropriate point to conserve resources. They indicated that families felt confused and distressed about this hospitalisation process. Fear of not being able to visit family members in hospital was preventing some families from admitting their ill relatives to hospital (both patients with and without COVID-19). There was a perceived lack of information on restrictions around visiting by family and religious leaders. Bedside ministry was seen as crucial for some faith communities, and religious leaders are currently continuing this work while provided with personal protective equipment. Many non-Christian patients were not used to accessing hospital chaplaincy services and had particular anxieties about costs and personal preferences. Although NHS workers were facing the extra burden of providing companionship and spiritual support to the dying, especially when admissions were highest, there was a gratifying sense of generosity across and between faith officiants and NHS staff. People were concerned that last wishes and wills occurring at the bedside or via telephone call would not be legally binding.

### Disposal and release of the body

Community leaders perceived that a safe death for the government involved the bodies of COVID-19 fatalities being disposed of quickly, without exposing others and favourably through cremation. They indicated cremation is unacceptable to some Christian, Muslim and Jewish communities. Even some people of no faith had strong reasons for preferring burial. The possible enforcement of cremation caused significant anxiety and could lead to social disturbances. Delayed release of the body was unacceptable for some Sikh, Hindu, Muslim and Jewish communities, but preferable to cremation for some Christian communities.

### Funerals

Banning funerals was unacceptable to most communities. At the time of research, funeral directors and crematoria were identified as the pinch points determining whether a deceased person was to get any officiant or attendees at a funeral. While it is traditional in many communities to host large funerals, smaller funerals can be experienced positively as being more ‘intimate’ occasions. The bereaved were distressed by a perceived inconsistency on the part of funeral parlours and local authorities regarding the permitted numbers of congregants, and restrictions on permitting family to view, wash and carry the body. Funeral attendees indicated that they found themselves torn between adherence to social distancing measures and their desire to physically comfort each other. The cost of funerals was a significant source of anxiety for some communities where existing government grants are not sufficient to cover funeral costs, leaving people reliant on credit or forced to opt for a ‘pauper’s funeral’ which was perceived as not allowing for a dignified burial process. Livestreaming or recording funerals and mourning ceremonies and private prayer meetings facilitated by technology were adaptations being implemented across communities.

### Mourning

It was said to be of the utmost importance that families felt able to ‘honour’ their dead to avoid, what one participant called, ‘complex grieving’, where the bereaved were left feeling guilt or unable to find a sense of closure. Normal practices of visiting, caring and cooking for the bereaved could not occur due to social distancing guidelines, leaving mourners isolated—though visiting became possible in some instances as restrictions eased. This is particularly acute for those who are unable to use, or cannot afford internet and telephone, and those whose fluency in English is insufficient for them to take advantage of remote counselling. It was indicated that communities were likely to interpret their experiences through cultural and historical lenses, and as such excess death and the denial of a funeral can trigger associations with traumatic events from the past, for example, the Holocaust of World War II in the Jewish community, and slavery in the Caribbean community.

## Collective loss

### National loss

This study showed that community leaders saw the pandemic as a traumatic period of national loss that transcended ethnic or religious boundaries. Though this event has been prolonged over two calendar years, its acuity was equivalent to that in the context of war, civil strife, terrorist events and large-scale accidents. However, it was clear that this loss was being disproportionately experienced and acknowledged in media discourse. As already mentioned, certain communities were suffering higher levels of mortality, exacerbating existing forms of deprivation. As Kokou-Kpolou *et al* suggest, dealing with collective loss involves essential processes of memorialisation.^40^ Yet, the fact that these lives have not been collectively memorialised thus far, as a matter of political priority, causes one to question if such lives are considered less ‘grievable’ than others in light of their marginal class, racial or ethnic position in the national imaginary.^41^ Now and in future crises, efforts should be made to acknowledge the disproportionate impact of mortality on particular communities through public communications and financial aid to adversely affected groups. A key ritual element of this plan would be to hold personalised communal memorials at a later date, for instance, a community-wide memorial service in a place of worship or community centre; and a national day of mourning. This allows for public recognition of the traumatic context of these deaths, and the acknowledgement of disproportionate loss which is crucial for long-term mental health management and social cohesion.^42^ There must also be a plan for the management of the impact on ‘involved staff’—medical staff, paramedics, funeral directors, ministers—some of whom are likely to experience post-traumatic stress disorder.

### Individual and community loss

This study showed that community leaders expressed a strong desire to have the dignity of death preserved during the COVID-19 pandemic.^43^ The institution of new rituals concerning dying, death and the funeral is feasible and will be generally accepted, but only if such rituals are seen as ‘authentic’ and, where possible, are based on existing rituals. It pointed to the urgent need for flexible government regulations that enable disparate cultural, religious and class communities to carry out their core practices—a response that might be institutionalised to deal with future crises. Preparation for hospital admission and possible death could be achieved by trusted figures advising people about how they would like the dying process to unfold. More generally, people could be encouraged to prepare their wills and write letters to their families conveying their wishes. However, a balance needs to be achieved between assisting with preparation for possible death and ensuring that they do not suffer too much anxiety.

Uniform application of government regulations across faith and non-faith groups is required in order to avoid feelings of cultural exclusion. Collaboration should be encouraged between faith and non-faith leaders, palliative care specialists and funeral directors to formulate and implement such flexible regulations. However, it is also essential to provide training and advice for the NHS and in Public Health Teams on the impact of stigma on health outcomes and on how to destigmatise interactions and communications in situations of care provision, release of the body and engagement with the bereaved. Consistency needs to be maintained across local authorities, mortuaries and funeral parlours on what ritual processes are permitted. However, local authority community engagement teams can make special efforts to acknowledge and understand the impact of mortality on adversely impacted groups. Direct financial help to those experiencing loss from COVID-19 deaths would be well received, especially for minority groups; while bereavement support services, in multiple languages and including culturally and religiously diverse staff, should be supported to provide psychosocial support via the conduits permissible during social restrictions.^44^

### Communication and consultation

This study revealed the importance of listening to the needs of communities in times of crisis and engaging local experts to build appropriate policies. Such infrastructures might be built to engage with communities in authentic ways prior to future crises. Respondents emphasised that they would experience the government as enabling a dignified dying process if it engaged in active consultations. Special effort should be made to include marginal communities, especially communities affected by poverty, minoritised communities and non-religious communities in developing, adapting and communicating policy guidelines around COVID-19 prevention, mourning processes and memorialisation. Consultation needs to take place with national and local-level organisations linked to local authority services, mutual aid groups and local resilience forums. The Community Champions scheme is an effective mechanism that can be used to reach minority ethnic communities, and provide non-stigmatising advice in relevant languages on multimorbidities and COVID-19 risks, vaccination access, lateral flow testing in schools, and workplace risks and mitigations.^45^ As Ryan *et al*^45^ observed in the case of the Ebola pandemic, trust, openness, reflexivity and accountability can strengthen effectiveness of emergency response.

## Conclusion

Across communities, a ‘good death’ in normal circumstances means allowing people to die in the company of loved ones or others who can provide spiritual support, ensuring that their bodies undergo appropriate ritual procedures and respecting their wishes regarding burial or cremation. Community leaders are willing to modify mourning processes in challenging circumstances in order to mitigate trauma for their constituents. At present, this means recognising those who have died during this pandemic should be prioritised as a matter of national and community importance. Provision of financial and psychosocial support to the bereaved is crucial, particularly in minority communities who have seen high mortality rates. Community recovery and trauma management in the coming years depends on such efforts to facilitate an honourable legacy for those who have died from COVID-19. Such insights should be institutionalised to avoid missteps and collective pain in future crises. The use of rapid ethnographic methods proves an effective methodology at rendering visible community responses and might be pursued with respect to other issues that arise in the pandemic and its aftermath.^46^ This is vitally important in complex, multifaith and multiethnic democracies in order to preserve social cohesion.

## Data Availability

Data are available upon request.
